# HMGB1 mediates microbiome-immune axis dysregulation underlying reduced neutralization capacity in obesity-related post-acute sequelae of SARS-CoV-2

**DOI:** 10.1038/s41598-023-50027-1

**Published:** 2024-01-03

**Authors:** Noelle C. Rubas, Rafael Peres, Braden P. Kunihiro, Nina P. Allan, Krit Phankitnirundorn, Riley K. Wells, Trevor McCracken, Rosa H. Lee, Lesley Umeda, Andie Conching, Ruben Juarez, Alika K. Maunakea

**Affiliations:** 1 Department of Biochemistry, Anatomy, and Physiology, University of Hawaiʻi at Mānoa, Honolulu, HI USA; 2 Deparment of Molecular Biosciences and Bioengineering, University of Hawaiʻi at Mānoa, Honolulu, HI USA; 3 Hawaiʻi Integrated Analytics, Honolulu, HI USA; 4 Deparment of Economics and UHERO, University of Hawaiʻi at Mānoa, Honolulu, HI USA

**Keywords:** Respiratory tract diseases, Inflammation, Microbiota, Cytokines

## Abstract

While obesity is a risk factor for post-acute sequelae of SARS-CoV-2 infection (PASC, "long-COVID"), the mechanism(s) underlying this phenomenon remains poorly understood. To address this gap in knowledge, we performed a 6-week longitudinal study to examine immune activity and gut microbiome dysbiosis in post-acute stage patients recovering from SARS-CoV-2 infection. Self-reported symptom frequencies and blood samples were collected weekly, with plasma assessed by ELISA and Luminex for multiple biomarkers and immune cell profiling. DNA from stool samples were collected at the early stage of recovery for baseline assessments of gut microbial composition and diversity using 16S-based metagenomic sequencing. Multiple regression analyses revealed obesity-related PASC linked to a sustained proinflammatory immune profile and reduced adaptive immunity, corresponding with reduced gut microbial diversity. In particular, enhanced signaling of the high mobility group box 1 (HMGB1) protein was found to associate with this dysregulation, with its upregulated levels in plasma associated with significantly impaired viral neutralization that was exacerbated with obesity. These findings implicate HMGB1 as a candidate biomarker of PASC, with potential applications for risk assessment and targeted therapies.

## Introduction

Although the number of new cases of SARS-CoV-2 infection globally is subsiding, the prevalence of post-acute sequelae of SARS-CoV-2 (PASC) in individuals recovering from COVID-19 remains a significant public health concern. In particular, individuals with pre-existing conditions associated with immune dysregulation are more susceptible to PASC^1^, and collectively, studies have associated systemic inflammation and reduced humoral activity with this condition^1,2^. In addition, given the role of the gut microbiome in regulating immune function, gut microbial dysbiosis also associates with PASC^3,4^. Further, obesity is a common risk factor for PASC in association with a dysregulated microbiome-immune axis^5^. Thus, the combination of metabolic dysfunction (from insulin resistance and adiposity) and pre-existing disruptions in the microbiome associated with chronic low-grade inflammation in obesity may exacerbate viral persistence that underlies PASC in recovering patients^6^.

PASC is a complex and multifactorial syndrome, and establishing its pathophysiology has proven challenging^7^, requiring further molecular assessment of implicated processes to distinguish its full biological cascade. PASC's molecular obscurity has prompted several challenges to disease management^5^. First, without molecular markers for laboratory testing, clinical diagnosis is the only option, likely leading to underdiagnosis. Additionally, without molecular diagnosis, there remains debate when defining and accepting the transition from COVID-19 to PASC^8–10^. Although biomarkers linked to COVID-19 severity and PASC have recently been proposed^11,12^, with no clear understanding of their relationship to PASC, these remain to be validated. Finally, a major challenge is the inability to predict who may develop PASC, which might otherwise facilitate prophylactic measures or therapeutic intervention to mitigate symptomatic onset^13^.

Herein, we explored immune- and gut microbiome-associated pathways in individuals recovering from SARS-CoV-2 infection in the post-acute stage to better characterize the development of PASC. We aimed to identify PASC susceptibility biomarkers as candidate PASC predictors and/or potential therapeutic targets. To molecularly resolve PASC, individuals recovering from COVID-19 were invited to participate in a 6-week longitudinal cohort study via weekly self-reporting their symptoms and providing blood samples. Plasma was subjected to enzyme-linked immunosorbent assays (ELISA) and Luminex technology to measure antibody response and inflammatory/metabolic biomarkers, while peripheral blood mononuclear cells (PBMC) were stained and FACS-sorted for immune cell profiling. Microbial DNA, extracted from a single stool sample obtained at the baseline timepoint (at study entry), was subjected to 16S-based sequencing using the Ion Torrent platform. Data was integrated and analyzed by Integrated Pathway Analysis (IPA) to identify potential PASC biological pathways, with candidate biomarkers cross-validated using regression analysis to assess their predictive potential.

In this cohort, we confirmed that obesity associated with increased frequency of PASC. Obesity-associated PASC consisted of a molecular profile of persistently elevated inflammatory responses, corresponding to reduced humoral activity and subsequent loss of viral neutralization. These features also associated with reduced microbial diversity, suggesting gut dysbiosis. Specifically, IPA identified the high mobility group box 1 (HMGB1) protein as a top pathway implicated by the data independent of body mass index (BMI), although HMGB1 response was perturbed in obesity. Stepwise pathway analysis of our data highlighted the role of the HMGB1 pathway in regulating the microbiome-immune axis in obesity-related PASC. Step-wise regression was used to determine the mechanism order beginning with obesity-associated modulation of the gut microbiome and its downstream effects on HMGB1 and viral neutralization. Significantly, baseline plasma levels of HMGB1 were predictive of neutralization capacity over the course of recovery, with high increased level with PASC symptoms. Importantly, our data demonstrate HMGB1 as a candidate biomarker for PASC with implications for developing prophylactic and therapeutic interventions.

## Results

### Recruitment and study cohort

We enrolled 48 COVID-19-recovered subjects residing in Oʻahu, Hawaiʻi between June and September 2020, excluding those with multiple follow-up absences. At the time of enrollment, no participants were vaccinated and most had recovered from mild symptoms, except for two obese participants who required hospitalization. The total cohort demographics are provided in Table 1. To explore the impact of obesity on PASC, we stratified participants into normal (BMI equal to or less than 24.9), overweight (BMI between 25 and 29.9), and obese (BMI equal to or greater than 30) groups, and assessed differences between these categories.

**Table 1:** The table presents demographic information for recovered COVID-19 individuals, further stratified by BMI groups.

	Total cohort	BMI group			P-value
		Normal	Overweight	Obese	
Participants (N; %)	48	14 (29%)	12 (25%)	22 (46%)	
Days since infection (mean ± SE)	44.24 ± 5.12	54.25 ± 9.89	46.83 ± 9.58	37.05 ± 7.64	0.13
Unknown	3	2	0	1	
Sex (N; %)	0.3				
Female	28 (58%)	10 (71%)	5 (42%)	13 (59%)	
Male	20 (42%)	4 (29%)	7 (58%)	9 (41%)	
Age (mean ± SE)	41.61 ± 3.37	44.93 ± 6.89	54.82 ± 5.47	31.53 ± 4.01	0.021
Age categories (N; %)	0.15				
0–18	8 (17%)	3 (21%)	0 (0%)	5 (23%)	
18–35	11 (23%)	2 (14%)	2 (17%)	7 (32%)	
35–50	12 (25%)	4 (29%)	3 (25%)	5 (23%)	
50–100	13 (27%)	5 (36%)	6 (50%)	2 (9.1%)	
Unknown	4 (8%)	0 (0%)	1 (8.3%)	3 (14%)	
BMI (mean ± SE)	30.06 ± 1.13	21.15 ± 0.80	27.71 ± 0.42	37.00 ± 1.05	< 0.001
A1c (mean ± SE)	5.24 ± 0.23	4.74 ± 0.09	5.33 ± 0.66	5.50 ± 0.33	0.093
A1c categories (N; %)	0.2				
Nondiabetic	43 (90%)	14 (100%)	11 (92%)	18 (82%)	
Prediabetic	0 (0%)	0 (0%)	0 (0%)	0 (0%)	
Diabetic	5 (10%)	0 (0%)	1 (8%)	4 (18%)	
Blood pressure (N; %)	0.2				
Low	4 (8.3%)	0 (0%)	1 (8.3%)	4 (14%)	
Normal	12 (25%)	7 (50%)	1 (8.3%)	4 (18%)	
Elevated	4 (8.3%)	1 (7%)	1 (8.3%)	2 (9%)	
Stage 1 hypertension	27 (56.3%)	6 (43%)	8 (67%)	13 (59%)	
Stage 2 hypertension	1 (2.1%)	0 (0%)	1 (8.3%)	0 (0%)	
Ethnicity (N; %)	0.015				
Asian	8 (17%)	5 (36%)	2 (17%)	1 (4.5%)	
NHPI^†^	18 (38%)	4 (29%)	2 (17%)	12 (54.5%)	
White	11 (23%)	3 (21%)	6 (50%)	2 (9%)	
Other	5 (10%)	2 (14%)	1 (8%)	2 (9%)	
Unknown	6 (12%)	0 (0%)	1 (8%)	5 (23%)	
Obesity biomarkers					
Leptin (ng/ml)	18.26 ± 2.28	10.56 ± 1.54	15.44 ± 3.05	24.96 ± 4.28	0.018
Unknown	3	1	0	2	
CRP (ng/ml)	65.40 ± 11.33	9.64 ± 2.87	74.48 ± 26.76	97.39 ± 16.57	< 0.001
Unknown	1	0	0	1	
Cellular phenotype					
Monocyte (%)	14.02 ± 1.80	8.70 ± 1.31	12.11 ± 1.10	17.55 ± 3.34	0.2
B cell (%)	3.27 ± 0.44	4.90 ± 1.05	3.51 ± 1.15	2.38 ± 0.31	0.068
T cell (%)	37.56 ± 3.16	42.14 ± 6.19	37.80 ± 6.14	35.27 ± 4.70	0.7
NK cell (%)	5.60 ± 0.71	5.02 ± 1.33	4.67 ± 1.34	6.36 ± 1.07	0.5
Unknown	10	5	2	3	

Categorical variables were tested using Fisher’s exact test and continuous variables were tested using Kruskal–Wallis one-way analysis of variance. ^†^*NHPI* Native Hawaiians and Pacific Islanders.

The time from acute infection period to study recruitment (day at entry) ranged from 10 to 101 + days (Supplemental Fig. 1) and average time was comparable across BMI groups, suggesting recovery time was not a covariate of obesity in this study. Ethnic composition varied across BMI groups as specified in Table 1. Age differences were observed across BMI groups, driven by the younger age of obese participants and the older age of overweight individuals. However, further compartmentalization of age into four categories reduced the statistical significance. Numbers of male and female participants varied across BMI categories although non-significantly. Regression modeling was conducted to assess the impact of age, sex, and ethnicity on BMI status in our cohort. Our model default parameters were set to male sex and Asian ethnicity. These potential covariates were not significant influences on BMI, suggesting these variables would not confound further downstream analyses (Supplemental Table 1).

Preliminary immune analyses were performed by profiling immune cell composition. Upon monoclonal antibody staining and sorting of PBMCs, marginally higher percentages of monocytes and lower percentages of B cells were observed in obesity, albeit non-significantly.

We assessed the presence of features associated with metabolic syndrome including blood pressure, type II diabetes status (determined by blood levels of A1c), and plasma levels of C-reactive protein (CRP) and leptin. CRP is a biomarker of systemic inflammation^14^ and circulating leptin is proportional to the amount of adipose tissue^15^, allowing both biomarkers to dually serve as molecular indicators of obesity. The incidence of hypertension and diabetic status increased with higher BMI, although group differences were non-significant. CRP and leptin were measured by ELISA using a CRP Human Instant ELISA™ Kit and Leptin Human Instant ELISA™ kit. We observed differences in CRP and leptin concentrations among groups, with elevated levels in the overweight and obese BMI groups compared to the normal BMI group.

Integration of obesity markers into our regression model revealed CRP was a covariate of BMI, independent of age, sex, and ethnicity. However, when the regression model was regenerated with CRP as the dependent variable, CRP levels were not related to age, sex, and ethnicity. This comprehensive analysis distinguished obesity as the key factor driving differences in our cohort and confirms the suitability of the cohort for studying obesity-related PASC.

### Data collection

**Figure 1:**
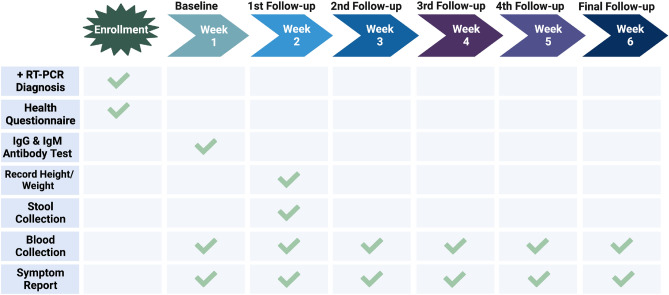
This figure presents a timeline and checklist for data acquisition in the study. The timeline shows the 7 key stages of the study, with each stage represented by a different color. The checklist provides an overview of the data points that were acquired and the methods used.

Figure 1 shows a schematic diagram of the data acquisition and study protocol. Study inclusion required proof of previous SARS-CoV-2 infection by RT-PCR testing, noninfectious status by a negative RT-PCR result at study entry, and a positive immunoglobulin G (IgG) and/or immunoglobulin M (IgM) antibody test at the first study visit. Participants completed a comprehensive health questionnaire regarding their overall health and potential comorbidities. Symptom reports were collected at each visit. Participant samples were collected weekly for a duration of 6 weeks; providing one stool sample for microbial sequencing after the first week of study entry, and blood samples every week, for separation into plasma and PBMCs.

### Obesity-related PASC is characterized by delayed symptom recovery and associate with attenuated humoral response

**Figure 2:**
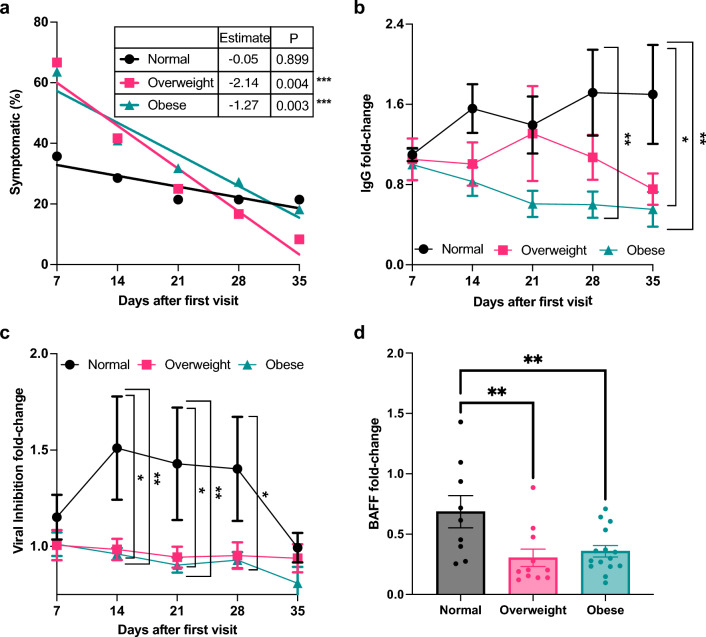
Decreased humoral response in obesity coincides with symptomology of post-acute sequalae of SARS-CoV-2. (**a**) Regression model of percentage of symptomatic participants as a variable of time per BMI group. (**b**) Ex-vivo assessment of Immunoglobin G in plasma plotted as IgG fold-change (mean and SEM) for BMI groups, two-way ANOVA with Tukey’s post hoc test. (**c**) Ex-vivo assay of viral neutralization capacity on plasma samples plotted as fold-change (mean and SEM), Sidak’s multiple comparison test. (**d**) Fold-change of B cell activating factor concentration from baseline to final follow-up for each BMI group, Sidak’s multiple comparisons test. *p < 0.05, **p < 0.01, ***p < 0.001.

The relationship between BMI and the average prevalence of PASC symptomology reported by each group is shown in Fig. 2a. At the first study visit, approximately 75% of overweight and obese participants reported symptoms, compared to less than 40% of those with a normal BMI. Symptomology decreased over time for all groups, eventually converging to similar levels by the final follow-up. Employing a longitudinal regression analysis framework, we found that normal weight participants exhibited a negligible estimated decline in symptom frequencies (coefficient = − 0.05). In contrast, overweight and obese participants exhibited a significant estimated decrease in symptom frequencies (coefficient = − 2.14, p = 0.004) and (coefficient = − 1.27, p = 0.003), respectively (Supplemental Fig. 2). Together, these data show distinctive symptomatic profiles, suggesting delayed recovery that was more pronounced in participants with higher BMI.

To understand the underlying factors contributing to delayed recovery, we first examined the correlation between antibody abundance and PASC symptoms within different BMI groups. Differences in generalized immunity were assessed by quantifying a crucial serum antibody, IgG, which plays a pivotal role in pathogen protection. Employing an anti-SARS-CoV-2 IgG ELISA test, relative changes in IgG levels were evaluated through log normalization of baseline IgG levels over the 6-week timeperiod. IgG concentrations of each participant were monitored weekly to calculate the average IgG fold-change with respect to BMI group. Differences in relative IgG fold-change among BMI groups were tested at each consecutive week (Fig. 2b). We observed a gradual decline in the abundance of anti-SARS-CoV-2 IgG in both overweight and obese groups when compared to participants of normal BMI. Specifically, the obese group exhibited significantly less IgG levels on day 28 (p < 0.01) and day 35 (p < 0.01) compared to IgG levels of normal BMI. Similarly, the overweight group exhibited significantly less IgG production on day 35 (p < 0.05) when compared to IgG levels of normal BMI. Together, the reduction of circulating IgG in participants with higher BMI suggests an attenuated maintenance of antibody production that is associated with delayed symptomatic recovery.

Analyte immunoassays for Human Coronavirus Ig were conducted to assess BMI-dependent humoral response specific to SARS-CoV-2 viral infection through evaluation of nucleocapsid, spike trimer, S1 subunit, and RBD antibody abundance (Supplemental Fig. 3a–d). Individuals of normal BMI maintained consistent levels of all four antibodies from baseline to final follow-up timepoints. Conversely, obese individuals exhibited a significant reduction from baseline to final follow-up in levels of nucleocapsid (p < 0.001), spike trimer (p < 0.05), S1 subunit (p < 0.05), and RBD (p < 0.01) antibodies. Overweight participants experienced a significant decrease in spike trimer antibody concentration (p < 0.05) from baseline to final follow-up, while maintaining stable levels for the other three antibodies. These data further highlight that inadequate maintenance of humoral responses are associated with increased BMI and correlate with the delayed recovery experienced by overweight and obese participants.

To explore the mechanism underlying reduced antibody maintenance and delayed COVID-19 recovery, we profiled viral neutralization capacity for all BMI groups by measuring relative levels of plasma-bound SARS-CoV-2 antibodies. We utilized a surrogate virus neutralization ELISA that mimics blocking of the receptor binding domain (RBD) of the SARS-CoV-2 spike glycoprotein and angiotensin-converting enzyme 2 (ACE2) human cell surface receptor. Neutralization capacity was compared across BMI groups by evaluating the relative fold-change in neutralization capacity over the study period. We plotted the average fold-change per week for each BMI group, testing for differences at each time point (Fig. 2c). Overall, normal BMI participants exhibited greater neutralization capacity than overweight and obese participants. Specifically, overweight individuals experienced significantly less neutralization capacity on day 14 (p < 0.05) and day 21 (p < 0.05), while obese participants experienced less neutralization capacity on day 14 (p < 0.01), day 21 (p < 0.01) and day 28 (p < 0.05) when compared to individuals of normal BMI. Longitudinal regression analyses revealed that obese participants had a significant decline in estimated neutralization capacity over time (coefficient = − 0.05, p = 0.003). Although normal and overweight BMI groups also exhibited an estimated decline in neutralization capacity this reduction was non-significant in both (coefficient = − 0.08, p = 0.109) and (coefficient = − 0.01, p = 0.56), respectively. Coupled with our findings above, the data suggests obesity-related PASC, experienced as delayed recovery from COVID-19 symptoms, is related to reduced antibody maintenance and decreased viral neutralization capacity.

To further examine the etiology of PASC, we noted that leukocyte populations have been shown to be perturbed, including the depletion of B cells^2^, which were marginally reduced in our overweight and obese groups. We thus examined B-cell activating factor (BAFF), a member of the TNF ligand superfamily recognized for its involvement in promoting B-cell survival and maturation^16^. Previous research has linked BAFF dysregulation in obesity^17^ suggesting it as a potential mechanism contributing to the diminished humoral response. We measured plasma levels of BAFF using the BAFF Human Instant ELISA™ Kit. Figure 2d shows the fold-change in BAFF concentration, comparing baseline to final follow-up BAFF levels for each BMI group. All BMI groups experienced a reduction in BAFF levels, indicated by a mean fold-change less than 1. However, overweight and obese participants experienced a more significant loss of BAFF levels when compared with normal BMI (p < 0.001) and (p < 0.001), respectively. Altogether, obesity-related PASC is distinguished by notable decreases in BAFF levels, consistent with the reduced B cell population we observed in obesity, contributing to the attenuated humoral response that is linked to delayed COVID-19 recovery.

### HMGB1 pathway is enriched in PASC and augmented in obesity-related PASC

To further molecularly characterize PASC and identify potential biomarkers, we adopted an agnostic approach to assess immunological changes by BMI group using a 65plex Luminex assay on baseline plasma samples. These data were integrated and analyzed for each BMI group separately using Ingenuity Pathway Analysis (IPA) software to predict enriched pathways relevant to the PASC phenotype for each BMI group. The top 10 enriched pathways reported by IPA and reflective of BMI categories are displayed in Table 2. Most enriched pathways were shared among BMI groups, suggesting individuals experienced similar pathophysiology during SARS-CoV-2 recovery that was independent of BMI.

**Table 2:** Top canonical pathways highlighted by IPA.

IPA canonical pathways	Normal BMI	Overweight BMI	Obese BMI
Pathogen induced cytokine storm signaling	+	+	+
IL-17 signaling	+	+	+
Airway pathology in chronic obstructive pulmonary disease	+	+	+
Macrophage classical activation signaling pathway	+	+	+
Granulocyte adhesion and diapedesis	+	+	+
Role of cytokines in mediating communication between immune cells	+	+	+
Multiple sclerosis signaling pathway	+	+	+
Wound healing signaling pathway	+	+	+
HMGB1 signaling	+	+	+
Agranulocyte adhesion and diapedesis	+	-	-
Differential regulation of cytokine production in macrophages and T helper cells by IL-17A and IL-17F	-	+	+

The table describes the overlap of enriched features according to BMI group. A positive (+) indicates the pathway is enriched for all individuals in the corresponding BMI group, while negative (-) indicates this pathway was not enriched in individuals of respective BMI groups.

**Figure 3:**
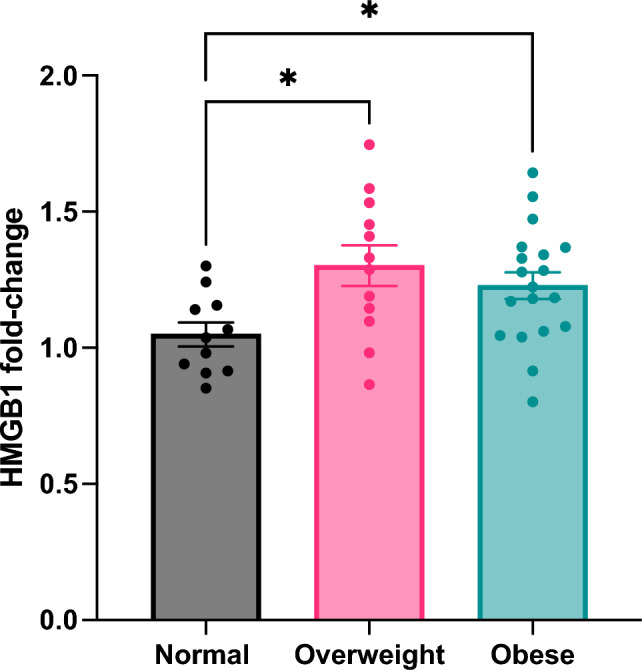
HMGB1 signaling is augmented in individuals with high BMI. HMGB1 fold-change from baseline to final follow-up stratified by BMI group, Sidak’s multiple comparisons test. *p < 0.05.

We further investigated the extracellular high-mobility group box 1 (HMGB1) pathway due to previous reports implicating this protein as a prospective biomarker and therapeutic target of COVID-19 severity^12^. HMGB1 is a central mediator of inflammation, generating the secretion of proinflammatory cytokines from dying and/or activated innate immune cells^11,18^. HMGB1 regulates both sterile and inflammatory responses^19^ by signaling through the receptor for advanced glycation end products (RAGE, a pattern recognition receptor)^20^ and toll-like receptors (TLR2 and TLR4), Using a colorimetric ELISA kit, we profiled HMGB1 protein levels at baseline and final follow-up to calculate fold-change in the plasma level of this protein over recovery (Fig. 3). HMGB1 levels remained consistent among normal BMI participants, while it significantly increased among overweight (p < 0.05) and obese BMI participants (p < 0.05). Upregulated HMGB1 levels among individuals with higher BMI suggests that perturbation of HMGB1 signaling is involved in PASC.

### Obesity-related PASC is associated with maintenance of hyper-inflammatory biomarkers

**Figure 4:**
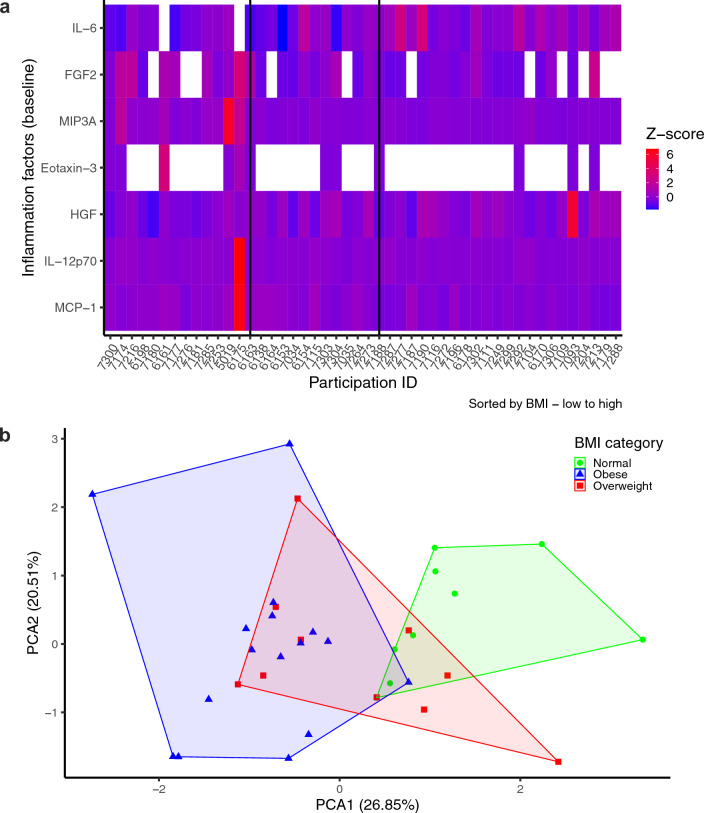
Baseline inflammatory profiles differ according to BMI group. (**a**) Heatmap generated from identified BMI-differentially abundant inflammatory markers at baseline timepoint. (**b**) Principal component analysis of differentially abundant inflammatory markers at baseline, grouped by BMI category.

**Figure 5:**
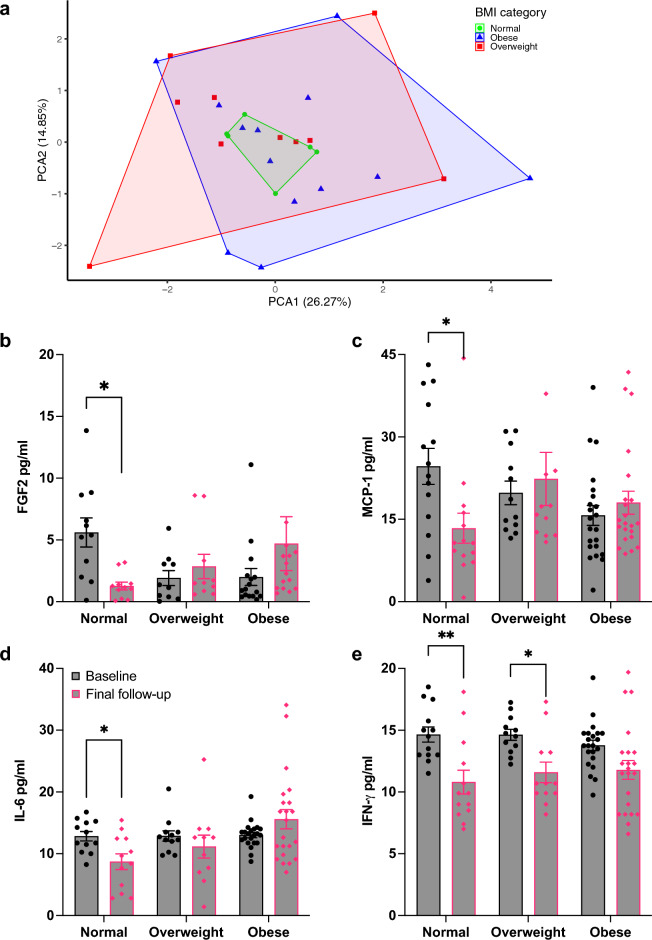
Inflammation is persistent in individuals with high BMI. (**a**) Principal component analysis of concentration changes in inflammatory markers from baseline to final follow-up, grouped by BMI category. (**b**–**e**) Ex-vivo assessment of inflammatory biomarkers at baseline and final follow-up, represented as mean with SEM for each BMI group, Sidak’s multiple comparisons test; *p < 0.05, **p < 0.01.

We hypothesized the increase in HMGB1 levels observed in higher BMI groups exacerbated chronic inflammation present in obesity due to increased adiposity. Adipose tissue, an endocrine organ, releases inflammatory cytokines, chemokines, and adipokines such as leptin that was elevated in overweight and obese individuals in our cohort^21,22^. Chronic inflammation fosters the proliferation of innate immune cell lineages leading to lymphocyte depletion and subsequent lymphopenia^23,24^. We investigated the presence of hyper-inflammation as an additional potential mechanism contributing to reduced humoral response and delayed recovery observed in obesity-related PASC. Differential abundance analysis of the 65plex Luminex assay revealed varying baseline concentrations of MCP-1, IL-12p70, HGF, Eotaxin-3, MIP3A, FGF2, and IL-6 in participants depending on BMI (Fig. 4a). Moreover, Principal Component Analysis (PCA) of this subset of immunological markers revealed patterns of BMI-clustering (Fig. 4b). A custom Luminex panel for 9 hyper-inflammatory cytokines was performed on final follow-up samples to assess inflammatory response by BMI group. PCA plots were generated using the change in concentration levels from baseline to final follow-up (Fig. 5a). When comparing responses across BMI categories, the tightly clustered normal BMI group demonstrated that individuals experienced a uniform response, with reduced concentrations of similar magnitude for all inflammatory biomarkers, a trend not observed among the overweight and obese BMI groups. We further characterized these cytokine dynamics from baseline to final follow-up according to BMI categories, which revealed BMI-dependent responses for FGF2, MCP-1, IL-6 and IFN-γ (Fig. 5b–e). Individuals of normal BMI exhibited a reduction in biomarker FGF2 (p < 0.05), MCP-1 (p < 0.01), IL-6 (p < 0.05), and IFN-γ (p < 0.01) concentrations. Overweight individuals had reduced IFN-γ (p < 0.05) levels, while obese individuals had persistent levels for all inflammatory biomarkers. These data suggest maintenance of hyper-inflammatory markers among individuals with higher BMI, which is associated with obesity-related PASC.

### Gut microbial dysbiosis correlates with PASC

**Figure 6:**
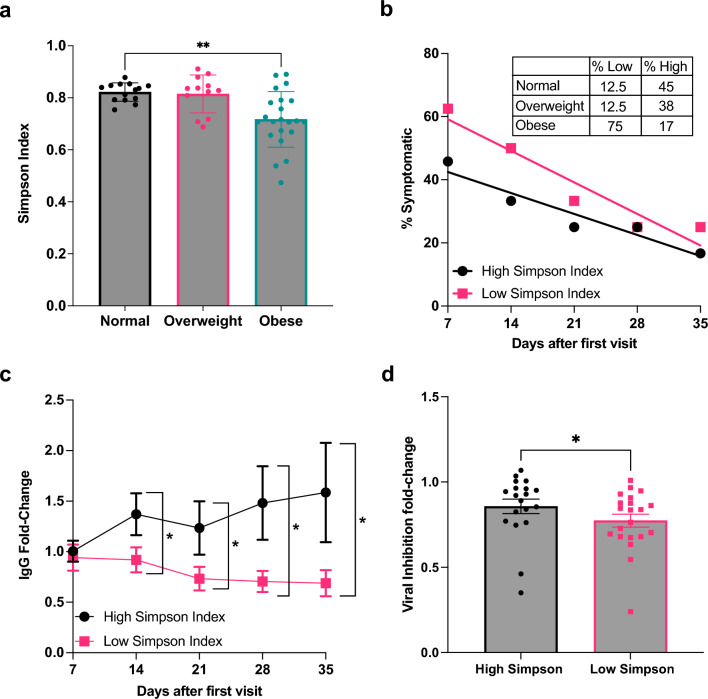
Reduced alpha diversity in obesity associates with symptomology of post-acute sequalae of SARS-CoV-2. (**a**) Simpson indices plotted according to BMI group, Kruskal–Wallis multiple comparisons test. (**b**) Regression model of percentage of symptomatic participants as a variable of time grouped into high and low diversity groups using upper and lower 50% cut-off. (**c**) Ex-vivo assessment of IgG in plasma plotted as fold-change (mean and SEM) for low and high diversity, one-way ANOVA with Tukey’s post hoc test. (**d**) Ex-vivo assay of viral neutralization capacity on plasma samples grouped by low and high diversity groups, plotted as fold-change from baseline to final follow-up (mean with SEM), Sidak’s multiple comparison test. *p < 0.05, **p < 0.01.

Overactivated inflammatory pathways linked to gut microbial dysbiosis have previously been implicated in PASC. Thus, we examined the degree to which microbial dysbiosis might account for the immune dysregulation we observed in our cohort by 16S-based metagenomics profiling. Supplemental Fig. 4 shows the relative abundance of phyla within the cohort and the phylum distributions between the BMI groups. ANOVA indicated altered composition across the BMI groups (p < 0.05), where the obese group exhibited reduced abundance of unclassified species. Given the lesser extent of unclassified species in obesity, we further examined the total species diversity using the Simpson index and evaluated relative diversity according to BMI group. Generally, higher diversity is related to robust immunity upon infection and overall better health outcomes^25,26^. Consistently, we observed that alpha diversity was significantly lower in the obese BMI group when compared to that of normal BMI (p < 0.01) (Fig. 6a). To explore the association among microbial diversity and obesity-PASC, individuals were regrouped based on the lower and upper 50% alpha diversity cut-offs. The average frequency of symptoms was recalculated for each diversity group and plotted longitudinally. The higher diversity group primarily consisted of normal and overweight individuals, accounting for 83% of this group. Conversely, the lower diversity group was predominantly composed of obese individuals, making up 75% of its composition. Initially, individuals in the upper diversity group displayed less frequent PASC symptomology (Fig. 6b), suggesting that higher BMI associates with reduced microbial diversity and is linked to the PASC phenotype. We conducted longitudinal regression analyses employing pairwise comparisons within both diversity groups to comprehensively examine the relationship between symptom recovery and BMI. Within the lower diversity group, we observed distinct coefficient estimates associated with different BMI categories. Specifically, for individuals with normal BMI, the coefficient estimate was − 0.71, for overweight BMI, this coefficient was − 6.43, and for obese BMI this coefficient was − 1.65. Notably, statistical significance was observed in this group for the relationships involving overweight BMI (p < 0.05) and obese BMI (p < 0.01). Conversely, within the upper diversity group, our analysis revealed distinct coefficient estimates as well. For those with normal BMI, the coefficient estimate was 0.13, for overweight BMI the coefficient was − 1.11, and for obese BMI the coefficient was 0.54. However, none of these relationships were statistically significant.

To relate microbial diversity to immunity in obesity-related PASC, we assessed normalized IgG levels to plot the relative fold-change for each diversity group by comparing the average change in IgG at each timepoint. We observed that lower microbial diversity corresponded with a decline in IgG maintenance over time. In the low diversity group, the loss of IgG maintenance ultimately resulted in significantly reduced IgG at day 14 (p < 0.05), day 21 (p < 0.05), day 28 (p < 0.05), and day 35 (p < 0.05) when compared to IgG levels among those individuals in the upper microbial diversity group (Fig. 6c). We also employed longitudinal regression analyses that involved pairwise comparisons within both diversity groups to gauge the relationship between microbial diversity and IgG maintenance. The lower diversity group exhibited a slight negative coefficient estimate of -0.07 while the upper diversity group had a positive coefficient of 0.11. However, neither of these findings reached statistical significance (Supplemental Fig. 5). Given these opposing trends, however, we calculated the fold-change in neutralization capacity from baseline to follow-up according to diversity group (Fig. 6d). Interestingly, we observed a steeper decline in the viral neutralization capacity among individuals in the low diversity group when compared to individuals with higher microbial diversity. Combined with the findings above, these results imply that elevated BMI can modulate the composition of the gut microbiome by influencing microbial diversity. These changes coupled with the observed attenuated antibody production and viral neutralization may altogether contribute to the slower recovery experienced more frequently, but not exclusively, by obese individuals.

### Pathway analysis shows baseline HMGB1 levels predict viral neutralization capacity

**Figure 7:**
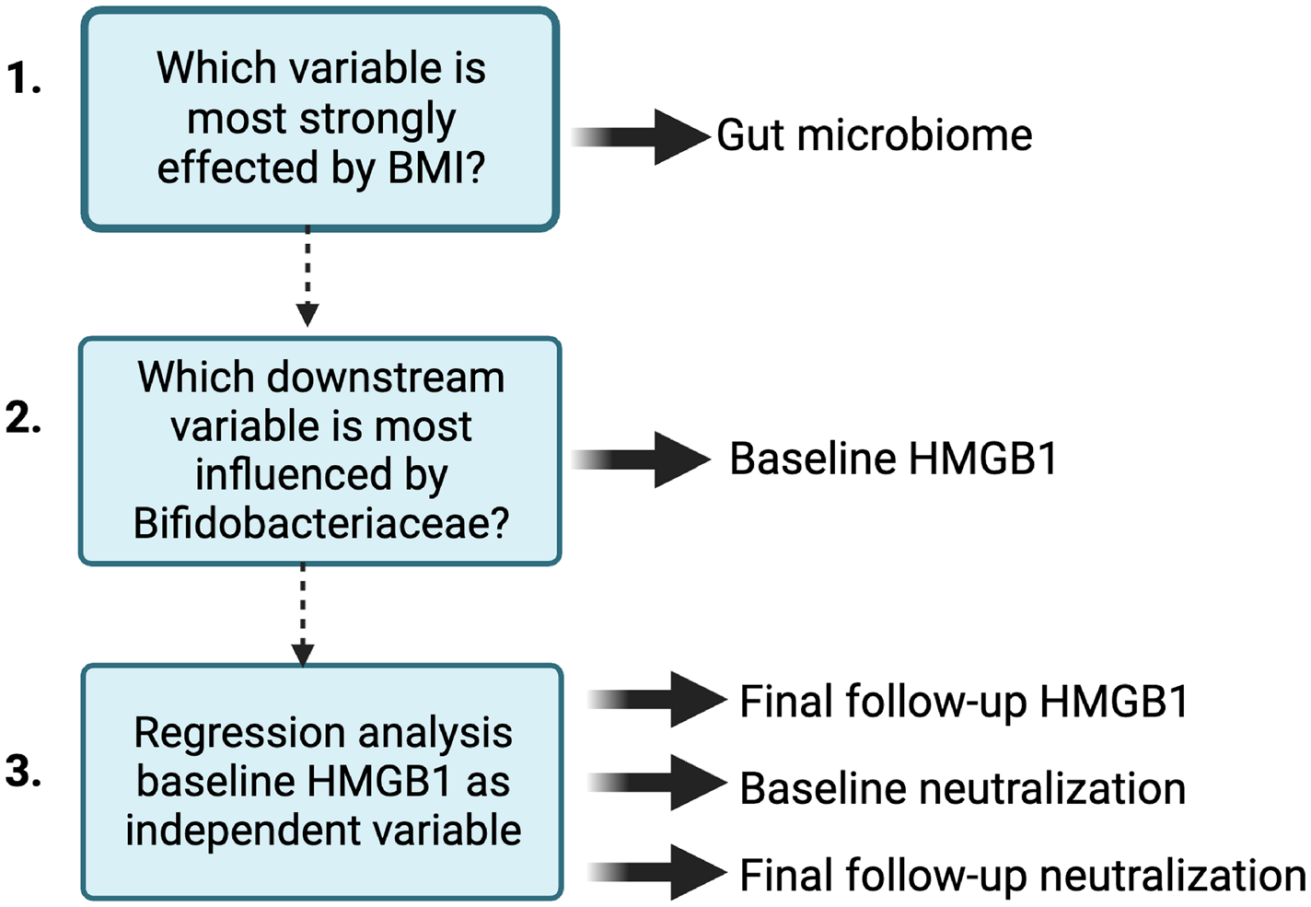
Workflow for identifying step-wise pathway analysis.

Although previous studies describe independent links between obesity, inflammation, the gut microbiome, and HMGB1, none show that these systems together influence PASC. In this study, we attempted to empirically identify a biological pathway to explain the obesity-associated attenuation of viral neutralization capacity we observed using a stepwise data-derived pathway analysis approach depicted by the workflow in Fig. 7. First, each variable was independently correlated to BMI, revealing associations between baseline viral neutralization capacity (p < 0.05), HMGB1 at final follow-up (p < 0.05), and various microbial abundances at the genus level (p < 0.05, Supplemental Table 3). The strongest association with BMI, microbial abundance, was therefore chosen as the first step in the pathway. While BMI-related genera did not directly influence neutralization, the *Bifidobacteriaceae* genus most strongly associated with baseline HMGB1 levels and was therefore chosen as the next step of the pathway. Baseline HMGB1 was used to predict final HMGB1 and viral neutralization capacity at both time points, using a multivariate statistical approach known as path analysis.

**Figure 8:**
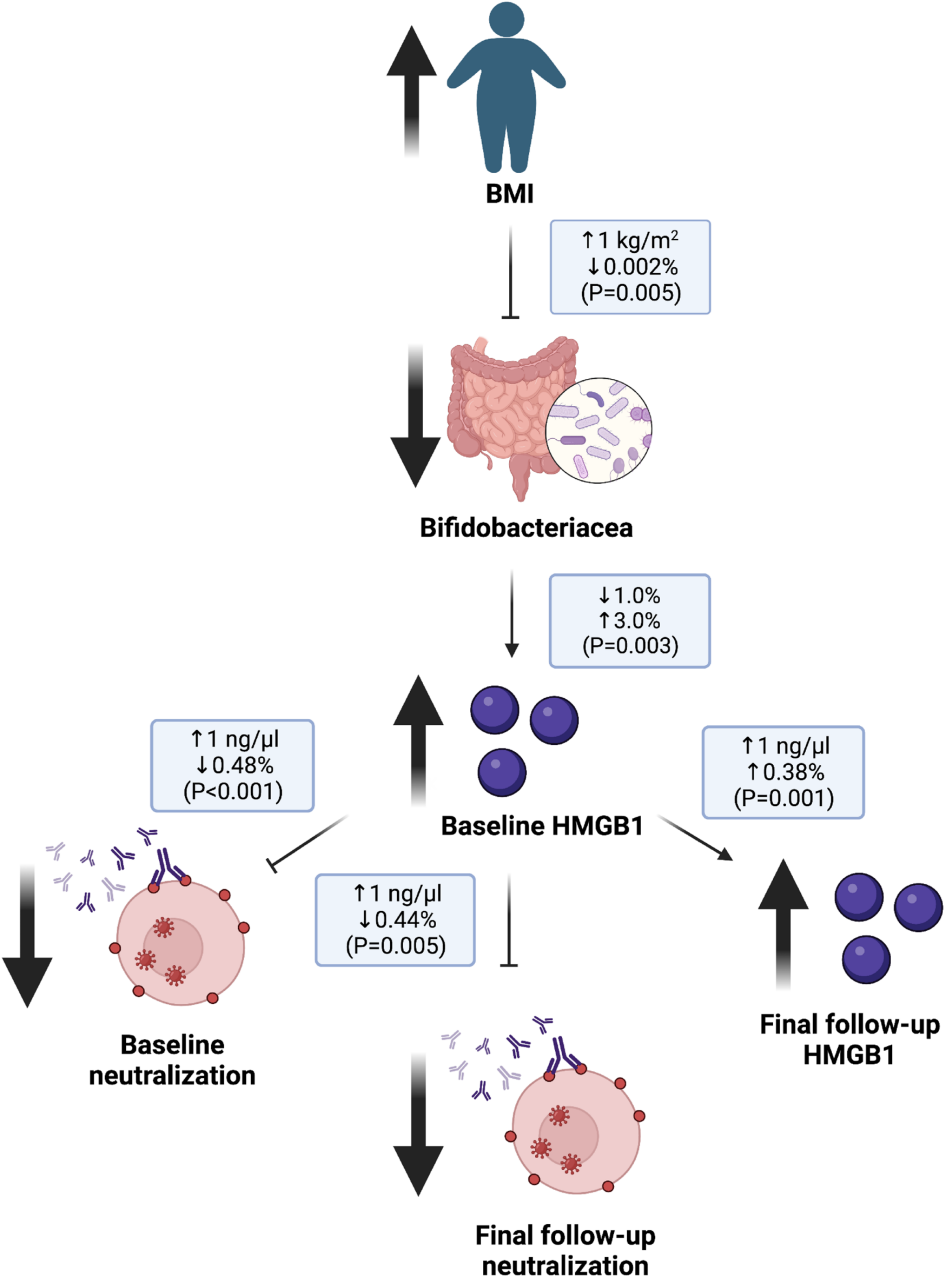
Data derived step-wise pathway analysis. Increased BMI influences post-acute sequelae of SARS-CoV-2 by reducing *Bifidobacteriacea* abundance. Modulation of the gut microbiome propagates overexpression of HMGB1 at baseline. This results in: (1) diminished viral neutralization capacity at baseline, (2) diminished neutralization capacity at final follow-up, and (3) elevated HMGB1 at final follow-up.

We further applied step-wise pathway analysis, conducted in the order identified by our data above, and discovered that BMI indirectly affects neutralization capacity (Fig. 8). Specifically, a one-unit increase in BMI associated with a 0.002% reduction in the relative abundance of the *Bifidobacterium* genus (p = 0.005), while a 1% reduction in *Bifidobacteriaceae* was associated with a 3% (p = 0.003) increase in HMGB1 concentration at baseline. Similarly, a one unit increase in baseline HMGB1 significantly associated with (1) 0.48% diminished baseline viral neutralization capacity (p < 0.001), (2) maintainance of elevated HMGB1 levels at the final follow-up by 0.38% (p < 0.001), and (3) a predicted loss of 0.44% of viral neutralization capacity at the final follow-up (p = 0.005). Following the delineation of our pathway, we conducted mediation analysis to investigate whether our step-wise variable relationships were mediated by a third variable. Three sets of simultaneous regression analyses, accounting for residual effects, were conducted to determine if incorporation of a third variable significantly influenced the path (Supplemental Table 4). No mediation effects were observed in the pathway relating BMI to viral neutralization capacity.

## Discussion

### The HMGB1 pathway influences the microbiome-immune axis, culminating in PASC

Obesity is known to increase PASC risk in COVID-19 patients^10^. To resolve the molecular basis of PASC in the context of obesity in our cohort, we first confirmed that delayed viral recovery (demonstrated by post-COVID-19 symptom frequency) associated with higher BMI. We then identified a molecular phenotype comprised of persistent systemic inflammation, loss of maintenance of humoral responses, and reduced diversity of the gut microbiome. Step-wise pathway analysis showed that initial HMGB1 levels predicted neutralization capacity at baseline and after 6 weeks during convalescence.

**Figure 9:**
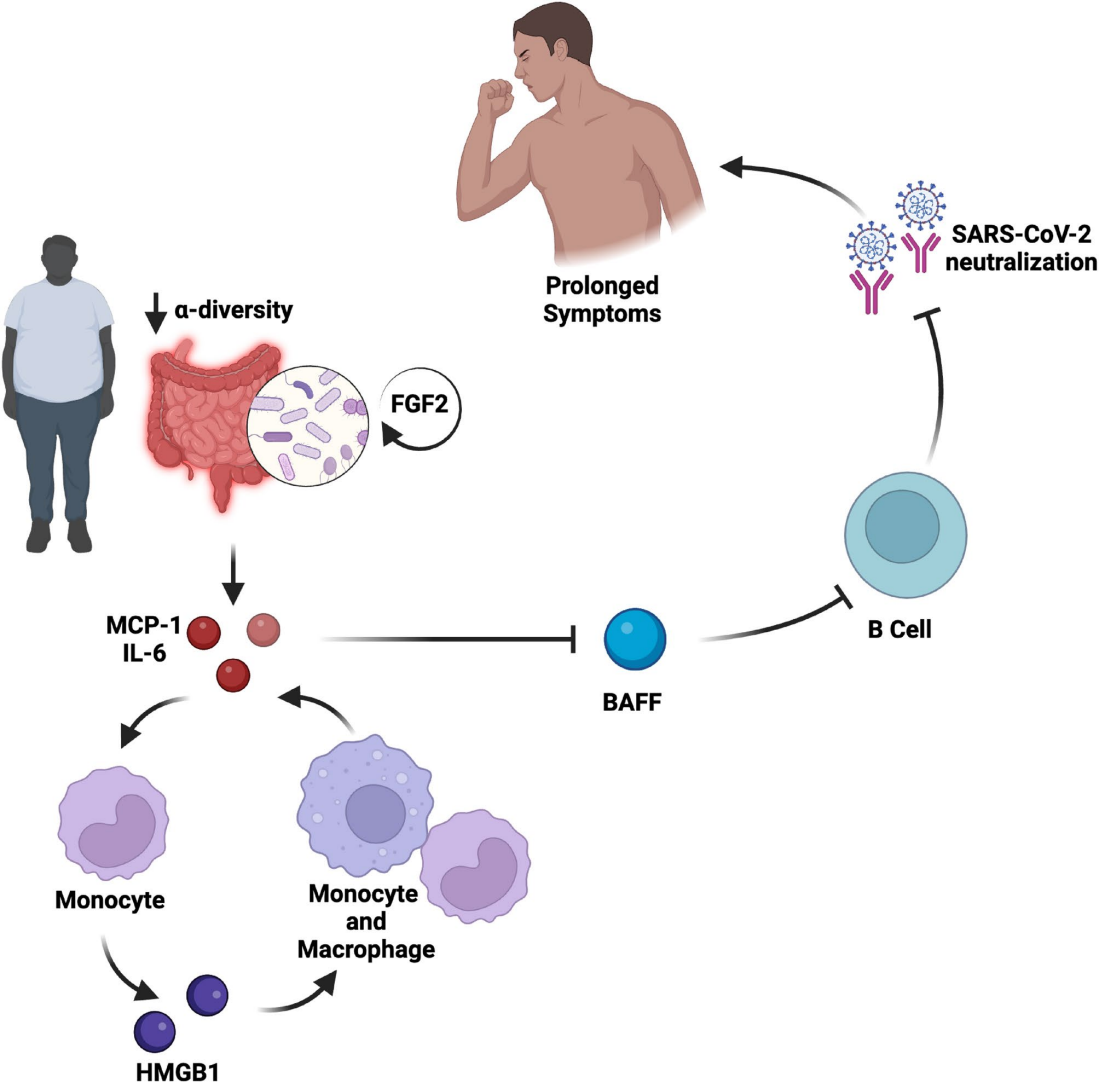
Proposed pathway for obesity-driven post-acute sequalae of SARS-CoV-2. Obese individuals experience microbiome dysbiosis, typically accompanied by intestinal inflammation. This triggers FGF2 production and IL-17, both known cooperate to promote repair of damaged epithelial cells in the intestine. Local intestinal inflammation primes individuals for pro-inflammatory responses, leading to a proinflammatory feedback loop from activated monocytes releasing HMGB1. This inflammatory signaling impedes BAFF maintenance and B cell propagation. In this reduced humoral state, SARS-CoV-2 viral infection evades the immune system experienced as reduced neutralization capacity. Compounded by a dysregulated microbiome, sustained inflammation and reduced humoral activity leads to prolonged symptoms and longer recovery.

Our molecular analysis revealed numerous biological pathways linked to PASC, revealing obesity-related reduced gut microbial diversity correlated with heightened inflammation (Fig. 9). Compared to those of normal BMI, the lower degree of α-diversity in the gut microbiome of overweight and obese individuals was associated with FGF2 expression and upregulated IL-17 (Fig. 5b and Table 2, respectively), known to promote repair of damaged epithelial cells^27^. Under this condition, persistent inflammatory signaling of MCP-1, IFN-γ, and IL-6 (derived reduced alpha diversity) further primes monocytes for pro-inflammatory activity. This initiates a proinflammatory feedback loop, upregulating HMGB1 excretion to further activate local and peripheral monocytes and macrophages. Prolonged inflammation inhibits BAFF expression (by inducing B cell apoptosis), thereby reducing antibody production and viral neutralization capacity upon SARS-CoV-2 viral exposure, and ultimately leads to prolonging acute COVID-19 symptoms (PASC). This PASC-promoting molecular cascade is particularly susceptible to obesity-mediated dysregulation. Our study highlights the confluence of obesity-associated microbiome dysfunction and immune responses in PASC, in which preexisting dysfunction contributes to a more dramatic inflammatory response to viral infection (thus reducing the effectiveness of the immune system). Our model (Fig. 9) suggests that this effect is exacerbated by upregulated HMGB1 due to various possible mechanisms, some of which we identified through pathway analysis.

In our cohort, obese individuals showed reduced microbial alpha diversity which has been linked to COVID-19 severity and PASC, irrespective of BMI^33,35,36^. While reduced microbial alpha diversity has been widely observed in obesity^28^, we also observed such dysbiosis, although less frequently, in non-obese individuals. This may be exacerbated by infection with SARS-CoV-2. In vivo mouse studies have shown that SARS-CoV-2 exposure alters gut microbial composition to reduce diversity, in unison with morphological changes that promote mucus and reduce the production of antimicrobial compounds^29^. In particular *Bifidobacterium* abundance has been extensively linked to human health due to its production of health-promoting short-chain fatty acids and protection against infection^30^, and we identified that reduced abundance of this genus most strongly influenced the obesity-related PASC phenotype, leading to reduced viral neutralization.

This study is not the first to link *Bifidobacterium* with SARS-CoV-2 infection. For example, loss of *Bifidobacterium* abundance has been linked to both COVID-19 severity^31^ and PASC existing 6-months post-infection^4^. However, we are the first to identify HMGB1 as a mediator of this association, as characterized in other contexts, e.g., where inoculation with probiotic *Bifidobacterium animalis* subsp. *lactis* BB-12 (BB12) decreased HMGB1 levels and partly ameliorated inflammatory responses following infection with the *Salmonella* Typhimurium strain LT2^32^. While our step-wise pathway analysis suggests that *Bifidobacterium* modulates HMGB1 reciprocally, HMGB1 may also indirectly modulate *Bifidobacterium* abundance in PASC pathology^33^.

In our study, COVID-19 recovering individuals with different degrees of microbial dysbiosis exhibited persistent inflammatory signaling of FGF2, MCP-1, IL-6, and IFN-γ. Microbial dysbiosis is accompanied by elevated lipopolysaccharides that promote inflammation, in part, by directly activating HMGB1 in resident immune and epithelial cells^34^. We observed this phenomenon in the marginal expansion of peripheral blood monocytes in patients with high BMI, a characteristic of obesity^35^. Moreover, these monocytes are known to be chronically activated. In addition to proinflammatory signaling, inflammatory monocytes can serve as reservoirs for viral replication and store increased viral titers^36^. For example, Patterson et al. recently discovered that individuals with severe COVID-19 and PASC, 15 months post-infection, had persistent SARS-CoV-2 S1 protein in non-classical monocytes^37^.

Under obesogenic conditions, elevated HMGB1 has been reported in adipose tissue acting as an alarm that leads to further HMGB1 induction^12,18,38^. HMGB1 signals through RAGE receptors, shown to be deleterious to the renin-angiotensin system by sustaining inflammation and oxidative stress involved in COVID-19 infection and multiorgan injury^39^ and possibly contributes to multi-organ PASC involvement. Moreover, ACE2, the SARS-CoV-2 entry receptor, is upregulated by HMGB1, creating a docking station and cache for the SARS-CoV-2 virus. When increased adiposity, HMGB1 overexpression, and SARS-CoV-2 reservoirs coexist, inflammation is persistently amplified. When accompanied by systemic inflammation, additional signaling from SARS-CoV-2 overburdens these processes and delays viral recovery. In that regard, Faizo et al. observed significantly reduced rates and titers of vaccine-induced spike-neutralizing antibodies in obese individuals^40^, while other recent studies showed that loss of neutralizing antibodies in obese COVID-19 patients with circulating autoimmune antibodies positively correlated with CRP levels and COVID-19 hospitalizations^41,42^. Although we did not expand upon the accumulation of autoantibodies in our cohort, these studies along with our data further indicate that deficiencies in adaptive immunity are inflammation-mediated and impacted by adiposity.

Altogether, our findings show that increased HMGB1 pathway signaling in obesity impairs adaptive immune response to SARS-CoV-2, contributing to the development of PASC with persistent chronic effects. Our findings suggest HMGB1 as a potential predictor of PASC risk and candidate for disease mitigation. However, our study was inherently limited by the small sample size, primarily due to attrition during the study period. This limitation constrained our ability to fully control for potential cofounders, including age and sex, as effectively as larger studies might have allowed. While our study cohort was powered to examine molecular mechanisms of PASC within the context of BMI stratification, it may not represent the full spectrum of PASC. Despite this limitation, our findings provide a valuable perspective on potential underlying mechanisms and provides a foundation for quantitative diagnosis. Future research with larger sample sizes and enhanced control for possible confounders is essential to validate and expand upon our findings.

## Materials and methods

### Human study participants

The protocol for this study was approved by the University of Hawaiʻi Office of Research Compliance 2019-00376 and performed in accordance to said guidelines. Informed consent was obtained from all participants and/or their legal guardians. Recruitment was advertised through flyers posted at the University of Hawaiʻi at Mānoa and media outlets. Data were collected from Oʻahu residents who tested positive for COVID-19 at any time during the recruitment period, but not longer than three months prior to the first data collection. Participants were eligible for the study if they provided proof of a previous positive nasopharyngeal RT-PCR test but were currently non-infectious as deemed by a negative RT-PCR result. Initially, 71 participants were enrolled between June 1, 2020 and September 25, 2020. Following enrollment, confirmation of a previous SARS-CoV-2 infection was determined by specific IgG/IgM rapid antibody testing, at the first visit, by a finger blood prick. A positive result for SARS-CoV-2 IgG or IgM antibodies was necessary to advance the into the study; five participants were excluded due to negative antibodies. During the first visit, participants completed a health questionnaire, provided demographic data, and received a stool collection kit. To ensure privacy, all participants, and their respective survey responses were anonymized using a unique numerical ID. Participants were asked to return weekly for five additional follow-up visits. During the second follow-up, subjects submitted a stool sample and recorded their height and weight, for BMI calculation by dividing weight in kilograms by the square of height in meters. At each of the five follow-up visits, participants provided a blood sample and reported symptoms such as fever, cough, difficulty breathing, fatigue, muscle or body aches, headache, new loss of taste or smell, sore throat, congestion or runny nose, nausea, and diarrhea. Symptom percentages for each follow-up visit were calculated by summing the number of participants experiencing any number of symptoms, in that week, divided by the total number of participants having no symptoms during that week. Symptoms were deemed resolved if no longer reported during any of the follow-up visits. Individuals who missed more than two appointments were excluded from the study. A total of 48 participants were included in the final analysis. All participants received monetary incentives after each weekly appointment.

### IgG/IgM antibody detection test

Rapid patient screening for SARS-CoV-2 specific antibody tests was performed using RayBio® Coronavirus (SARS-CoV-2) IgM/IgG test kits (Colloidal Gold Method) (RayBiotech, Peachtree Corners, GA, USA, cat #CG-CoV-IgM, CG-CoV-IgG) to confirm COVID-19 detection in whole blood from a finger prick.

### Blood processing

Blood was processed within 24 h of collection to separate plasma and PBMCs using PBMC isolation tubes (STEMCELL Technologies, Vancouver, BC, Canada). Plasma samples were kept at – 80 °C, and PBMCs at − 150 °C, until ready to assay.

### Stool sample DNA/RNA extraction

DNA and RNA were extracted from stool samples using AllPrep® PowerFecal® DNA/RNA Kits (Qiagen. Inc., Valencia, CA, USA). From each sample, 1–2 μl of purified DNA and RNA were analyzed using a NanoDrop™ Microvolume Spectrophotometer (Thermo Fisher Scientific, Waltham, MA, USA) to assess sample quality, in addition to a Qubit™ Fluorometer (Thermo Fisher).

### 16S rRNA sequencing and analysis

An Ion Torrent 16S™ Metagenomics Kit (Thermo Fisher) was used to amplify 16S rRNA genes of all stool samples. Following the amplification conditions indicated in the manufacturer’s protocol, PCR was performed in two pools, each containing specific primer sets. After PCR amplification, equal volumes (20 µL) of PCR products from pools 1 and 2 of each sample were combined into a single PCR tube. Then, 40 µL of the combined PCR product was purified using the Agencourt Ampure XP kit, according to the manufacturer’s instructions (Beckman Coulter, Brea, CA, USA). DNA was quantified using the Qubit Fluorometer with the Qubit dsDNA BR Assay Kit (Thermo Fisher Scientific, Warrington, England), and the DNA library was prepared using the Ion Plus Fragment Library Kit (Thermo Fisher Scientific, Austin, TX, USA) and Ion Xpress Barcodes Adapters 1–80 Kit (Life Technologies, Carlsbad, CA, USA) according to the manufacturer’s protocol. Each library was diluted to a concentration of 80 pM and equal volumes of each library were pooled. The Ion 510™ & Ion 520™ & Ion 530™ Kit (Thermo Fisher Scientific, Austin, TX, USA) was used to clonally amplify the pooled library of nanosized ionosphere particles by emulsion PCR. Bead enrichment and chip loading were conducted using an Ion Chef Instrument. The parallel sequencing reaction was performed using the Ion S5 Next-Generation Sequencing system with Ion 530 chips.

After obtaining sequencing reads (Supplemental Table 2), we performed rarefaction at n = 10,000, on microbial absolute abundance, to eliminate sample bias from uneven counts, further excluding six samples, to leave 42 for further analysis. The results were analyzed using the Ion Reporter Software on the Ion 16S Metagenomics Kit analysis module. In the scope of analysis, the reads were mapped to two reference 16S rRNA databases, Greengenes v13.5 and MicroSEQ ID v3.0, following the manufacturer’s instructions. Chimeric sequences were automatically identified and removed using Ion Reporter software^43,44^. To cluster the sequences, operational taxonomic unit (OTU) analysis was performed using Ion Reporter Software, which runs Quantitative Insights into Microbial Ecology (QIIME). Alpha and beta diversity graphics created by QIIME software were exported from Ion Reporter Software. For alpha diversity analysis, the observed species and Shannon, Fisher, and Simpson indices were generated to analyze species diversity within samples.

### Antibody detection ELISA

The SeroFlash™ SARS-CoV-2 IgG/IgM ELISA Fast Kit (Epigentek Inc., Farmingdale, NY, USA) was used to quantify SARS-CoV-2 spike protein IgM and IgG antibodies in plasma samples. For further confirmation and characterization, the Human Coronavirus Ig Total 11-Plex ProcartaPlex™ Panel (Thermo Fisher) was used for screening of four anti-SARS-CoV-2 antibodies (spike trimer, S1 subunit, receptor binding domain (RBD), and nucleocapsid), and six anti-coronavirus strains (SARS-CoV-1, MERS, CoV-NL63, CoV-KHU1, CoV-229E, and CoV-OC43). Fluorescent signals were analyzed using a Luminex 200 instrument (R&D Systems, Inc., Minneapolis, MN, USA), and data processed using Bio-Plex Manager™ software (Bio-Rad Laboratories, Inc., Hercules, CA, USA).

### Inflammation factors concentration measurements

#### Inflammatory and metabolic biomarkers

Exploration of immunological biomarkers at the baseline timepoint was performed for plasma cytokine concentrations of G-CSF (CSF-3), GM-CSF, IFN alpha, IFN gamma, IL-1 alpha, IL-1 beta, IL-2, IL-3, IL-4, IL-5, IL-6, IL-7, IL-8 (CXCL8), IL-9, IL-10, IL-12p70, IL-13, IL-15, IL-16, IL-17A (CTLA-8), IL-18, IL-20, IL-21, IL-22, IL-23, IL-27, IL-31, LIF, M-CSF, MIF, TNF alpha, TNF beta, TSLP; chemokines BLC (CXCL13), ENA-78 (CXCL5), Eotaxin (CCL11), Eotaxin-2 (CCL24), Eotaxin-3 (CCL26), Fractalkine (CX3CL1), Gro-alpha (CXCL1), IP-10 (CXCL10), I-TAC (CXCL11), MCP-1 (CCL2), MCP-2 (CCL8), MCP-3 (CCL7), MDC (CCL22), MIG (CXCL9), MIP-1 alpha (CCL3), MIP-1 beta (CCL4), MIP-3 alpha (CCL20), SDF-1 alpha (CXCL12; growth factors FGF-2, HGF, MMP-1, NGF beta, SCF, VEGF-A and soluble receptors APRIL, BAFF, CD30, CD40L (CD154), IL-2R (CD25), TNF-RII, TRAIL (CD253) and TWEAK, using a human Immune Monitoring 65-Plex Human Procarta™ Plex Panel (ThermoFisher part no. EPX650-10065-901), according to the manufacturer’s instructions. A smaller custom panel was used to investigate immunological markers at time of final follow-up. This panel included IFN gamma, IL-1 beta, IL-6, IL-8, IL-10, MCP-1, TNF alpha, VEGF-A, and Insulin (ThermoFisher part no. PPX-09, Assay ID: MXEPTU6) and was performed according to the manufacturer’s instructions. For both assays, samples were centrifuged at 13,000×*g* for 2 min to pellet the aggregates, with a standard curve generated using the manufacturer's antigen standards. Bead counts below 35 were insufficient for the analysis and were excluded from the analysis. Fluorescent signals were analyzed using a Luminex 200 instrument (R&D Systems, Inc., Minneapolis, MN, USA). The Bio-Plex Manager™ software (Bio-Rad Laboratories, Inc., Hercules, CA, USA) was used for data processing.

#### Other biomarkers

To evaluate the general indications of inflammation, CRP plasma levels were measured using a Human CRP Instant ELISA Kit (Thermo Fisher Scientific, Inc., Waltham, MA, USA) according to the manufacturer’s instructions. The samples were diluted 1:500 with assay buffer before use. Samples that were above the standard curve were further diluted and assayed a second time. BAFF measurements were performed using the BAFF Human Instant™ ELISA Kit (catalog no. BMS2007INST Thermo Fisher), according to the manufacturer’s instructions. HMGB1 plasma levels were assessed using a colorimetric Elisa Kit (Part No. NBP2-62766, Novus Biologicals, Littleton, CO, USA), according to the manufacturer’s instructions, without sample dilution. All ELISA assays were performed using a SpectraMax ABS/ABS Plus Microplate Reader (Molecular Devices, San Jose, CA, USA). Leptin plasma concentrations were measured using a Leptin Human Instant™ ELISA kit (Thermo Fisher), according to the manufacturer’s instructions. Samples were diluted 1:25 with assay buffer before use.

### PBMC phenotyping

Viably cryopreserved PBMCs of approximately 1.0 × 10^7^ from individuals, at weeks 2 and 6 of our protocol, were first thawed in AIM-V serum-free media (Thermo Fisher) supplemented with 1:50 DNase (Sigma-Aldrich, St. Louis, MO, USA), washed, and resuspended in wash buffer (PBS, 3% BSA, and 1 mM EDTA). Aliquots of 1.25 × 10^5^ PBMCs were taken from each sample for flow cytometry-based cellular phenotyping assays, to determine cell type compositions. Cells were counted using a Countess Automated Cell Counter (Life Technologies, Carlsbad, CA, USA). Aliquots were stained with yellow amine fluorescent reactive dye (YARD; Thermo Fisher Scientific) and then with anti-CD16 Brilliant Violet 421 (Clone 3G8), anti-CD3 V500 (Clone UCHT1), anti-CD14 Qdot®605 (Clone TüK4), anti-CD56 Pe-Cy7 (Clone B159), anti-CD19 PE-Cy7 (Clone SJ25C1), anti-CD20 Pe-Cy7 (Clone 2H7), and anti-HLA-DR APC-H7 (Clone G46-6) to identify leukocyte subpopulation frequencies. Anti-CD16 was purchased from BioLegend, Inc. (San Diego, CA, USA). Anti-CD3, anti-CD56, anti-CD20, anti-CD19, and anti-HLA-DR antibodies were obtained from BD Biosciences (San Jose, CA). Anti-mouse Ig/Negative Control (FBS) Compensation Particle Set (BD Biosciences) was used for compensation analysis of fluorescent signals emitted by each fluorochrome from the multicolored cellular phenotyping panel employed. Anti-mouse Ig compensation beads were stained with fluorochrome-conjugated antibodies in separate wells. ArC™ Amine Reactive Compensation Bead Kit (ThermoFisher) reactive bead/negative beads were used to compensate for YARD (Live/Dead stain) fluorescent signals. Stained cells from PBMCs and compensation particles were analyzed using a 4-laser BD LSRFortessa flow cytometer (BD Biosciences). Data were analyzed using FlowJo software (Tree Star, Inc., Ashland, OR, USA). The frequency (%) of each cell type was determined using the event count (specific events/total events) with debris exclusion.

### Statistical analysis

Comparative analyses of clinical, immunological, cell flow cytometry, and qPCR data were performed using ANOVA Mixed-effects analysis with Sidak’s multiple comparisons test correction. Non-parametric tests for clinical, immunological, and cell flow cytometry data were used primarily because the majority of data were non-normally distributed, as measured using the D’Agostino and Pearson tests for normality. Graphing and statistical analyses were performed using Prism 9, version 9.0c (GraphPad Software, La Jolla, CA, USA). Statistical significance was set at p < 0.05.

Longitudinal data was analyzed by performing BMI or diversity grouped pairwise comparisons without controlling for any covariates. This approach adopted a participant-specific control strategy, utilizing each unique participant as their own control. By doing so, we aimed to minimize potential confounding effects stemming from unaccounted variables. The use of participant-specific controls helped to account for individual-level variability and fluctuations over time.

To predict associative outcomes, several stepwise multiple logistic regression models were tested for clinical features, immunological features, and qPCR results that were identified by Wilcoxon Rank-Sum tests followed by multiple testing corrections (FDR-adjusted p < 0.01). In the order identified by stepwise analysis, we applied a multivariate statistical approach known as path analysis to carry out the predictive analysis. Specifically, we utilized the lavaan R package for latent variable analysis, accommodating for independent variables in one equation that may be potential dependent variables in another.

The mediation analysis was conducted using the Lavaan R package, commonly used for structural equation modeling and analysis^45^. Our analysis involved the estimation of three simultaneous sets of regression equations that model the direct effect, mediator effect, indirect effect, and total effect by accounting for residual errors.

A t-test was performed to assess the similarity between the overall (n = 48) and rarefied (n = 42) gut microbiome datasets. Correlation analysis was used to assess the association between the relative abundance of the gut microbiome and BMI. Moreover, path analysis was used to shape the research question of how differences in BMI impact PASC.

### Ingenuity pathway analysis (IPA)

The differential expression of cytokines, chemokines, and growth factors was used as an input for the Ingenuity Pathway Analysis (IPA) (Qiagen, United States). The core analysis module in IPA was used to deduce differentially regulated canonical pathways, upstream regulators, diseases and biological functions, and novel gene networks based on Fisher’s exact test (p-value cutoff at 0.05).

### SARS‐CoV‐2 surrogate virus neutralization test (sVNT)

The surrogate virus neutralization test** (**GenScript, NJ, USA) is a blocking ELISA that mimics the virus neutralization process, detecting circulating neutralizing SARS‐CoV‐2 antibodies that block the interaction between the RBD and ACE2 on the cell surface receptor of the host. The test was isotype- and species-independent. Plasma samples were diluted 10 × with sample dilution buffer and assayed according to the GenScript protocol. The absorbance of the sample was inversely dependent on the titer of the anti-SARS-CoV-2 neutralizing antibodies.

### Statement of ethics

This study and its protocol were approved by the University of Hawaiʻi Office of Research Compliance 2019-00376.

### Supplementary Information

Supplementary Information.

## Data Availability

Data is available upon reasonable request to AKM.
