# Author Correction: HMGB1 mediates microbiome-immune axis dysregulation underlying reduced neutralization capacity in obesity-related post-acute sequelae of SARS-CoV-2

**DOI:** 10.1038/s41598-024-56011-7

**Published:** 2024-03-19

**Authors:** Noelle C. Rubas, Rafael Peres, Braden P. Kunihiro, Nina P. Allan, Krit Phankitnirundorn, Riley K. Wells, Trevor McCracken, Rosa H. Lee, Lesley Umeda, Andie Conching, Ruben Juarez, Alika K. Maunakea

**Affiliations:** 1 Department of Biochemistry, Anatomy, and Physiology, University of Hawaiʻi at Mānoa, Honolulu, HI USA; 2 Deparment of Molecular Biosciences and Bioengineering, University of Hawaiʻi at Mānoa, Honolulu, HI USA; 3 Hawaiʻi Integrated Analytics, Honolulu, HI USA; 4 Deparment of Economics and UHERO, University of Hawaiʻi at Mānoa, Honolulu, HI USA

Correction to: *Scientific Reports* 10.1038/s41598-023-50027-1, published online 03 January 2024

The original version of this Article contained an error in the spelling of the author Trevor McCracken which was incorrectly given as Trevor A. McCraken.

The original article has been corrected.

