# Supplementary material for: HMGB1 mediates microbiome-immune axis dysregulation underlying reduced neutralization capacity in obesity-related post-acute sequelae of SARS-CoV-2
Source: Sci Rep. 2024 Jan 3;14:355. doi: 10.1038/s41598-023-50027-1 (PMC10764757; doi:10.1038/s41598-023-50027-1)
Supplement: Supplementary file 1 — Supplementary Information. [file 41598_2023_50027_MOESM1_ESM.pdf]

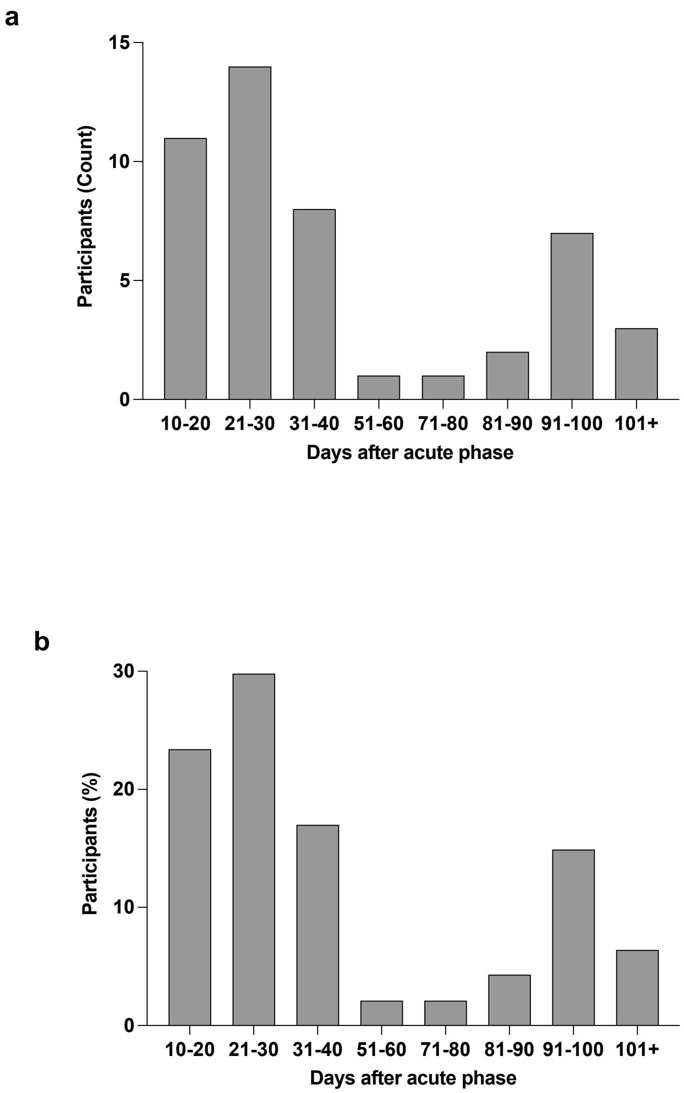

Supplemental Figure 1. Distribution of time since acute infection before participants enrolled in study. (a) Participants represented as counts. (b) Participants represented as percentages.

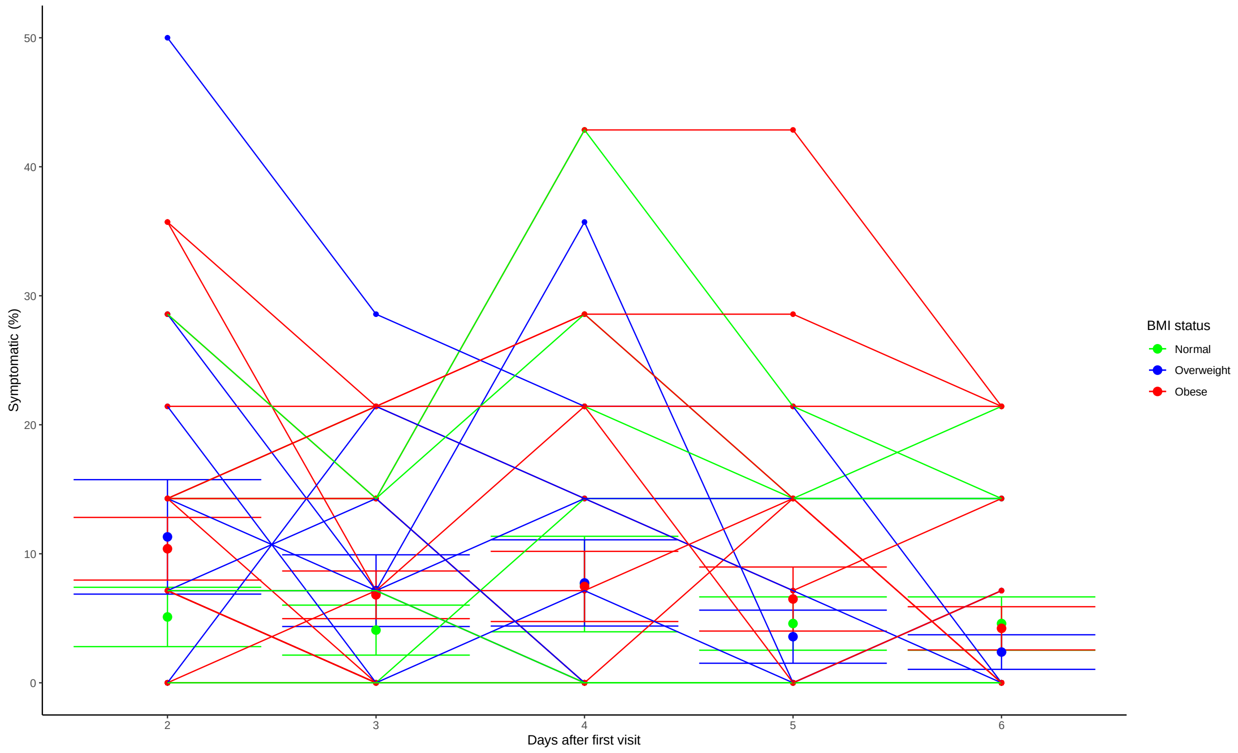

Supplemental Figure 2. Longitudinal Analysis of Symptom Prevalence Across BMI Categories. Pairwise regression models grouped by BMI categories. Coefficient estimates and statistical significance are as follows: normal BMI group (coefficient = -0.05, p = 0.899), overweight BMI group (coefficient = -2.14, p = 0.004), and obese BMI group (coefficient = -1.27, p = 0.003). $p<0.05$ is considered significant.

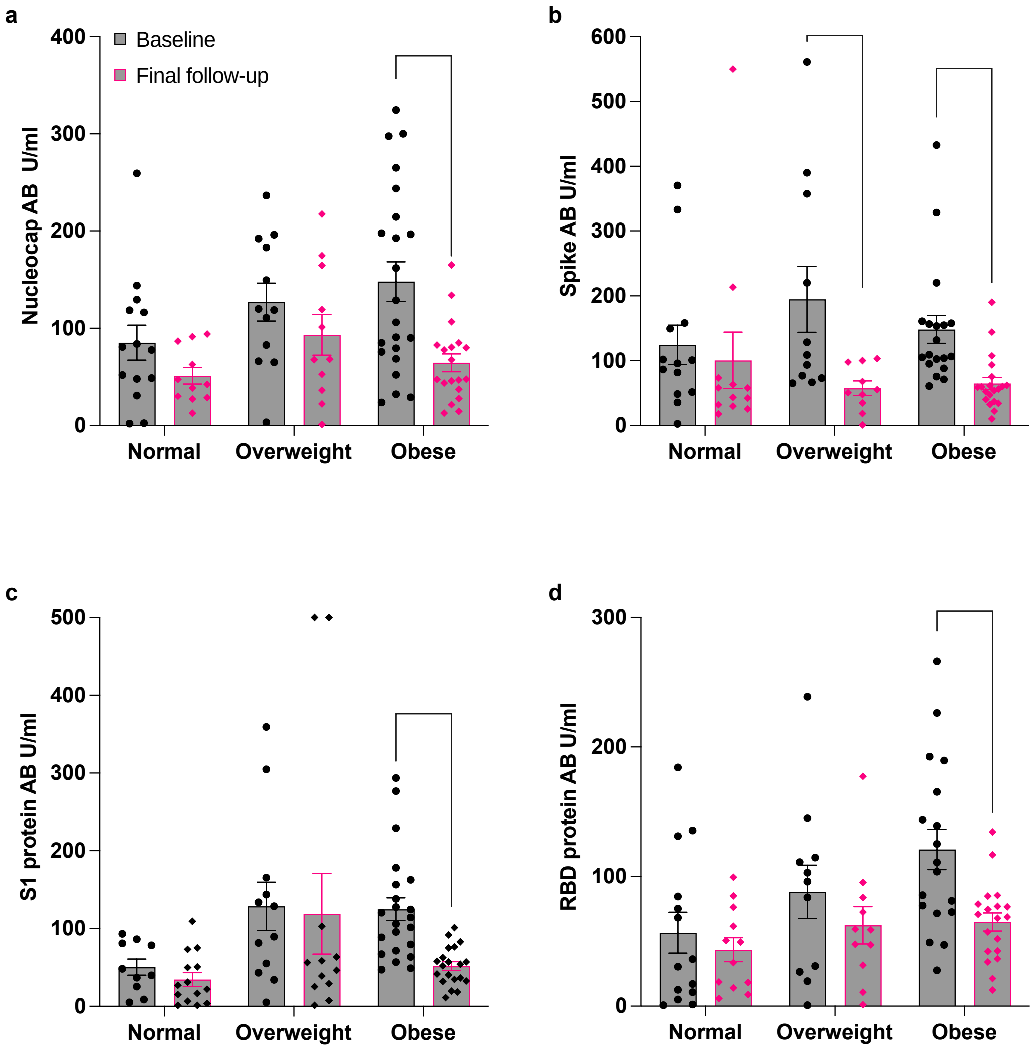

Supplemental Figure 3. Differences in SARS-CoV-2 antibody trajectories among BMI groups. (a) Nucleocapsid antibody levels (mean and SEM) at baseline and final follow-up for BMI groups, Sidak's multiple comparisons test. (b) Spike antibody levels (mean and SEM) at baseline and final follow-up for BMI groups, Sidak's multiple comparisons test. (c) S1 protein antibody levels (mean and SEM) at baseline and final follow-up for BMI groups, Sidak's multiple comparisons test. (d) Receptor Binding Domain antibody levels (mean and SEM) at baseline and final follow-up for BMI groups (n = 48), Sidak's multiple comparisons test. *p<0.05, ** p<0.01, *** p<0.001.

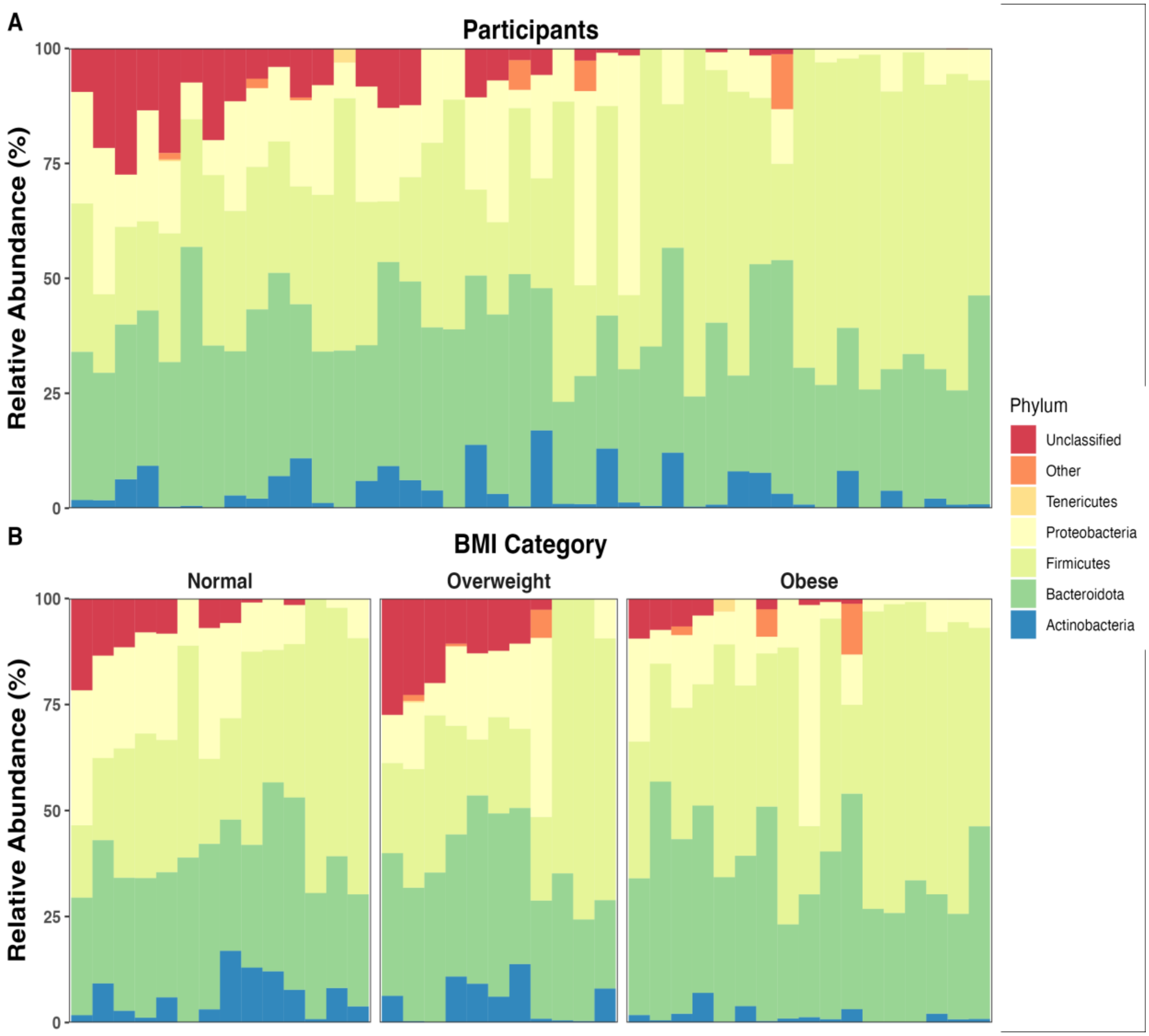

Supplemental Figure 4. Gut microbiome histogram of phylum relative abundance. Metagenomic 16s sequencing results for (a) total cohort composition and (b) Compositions grouped by BMI.

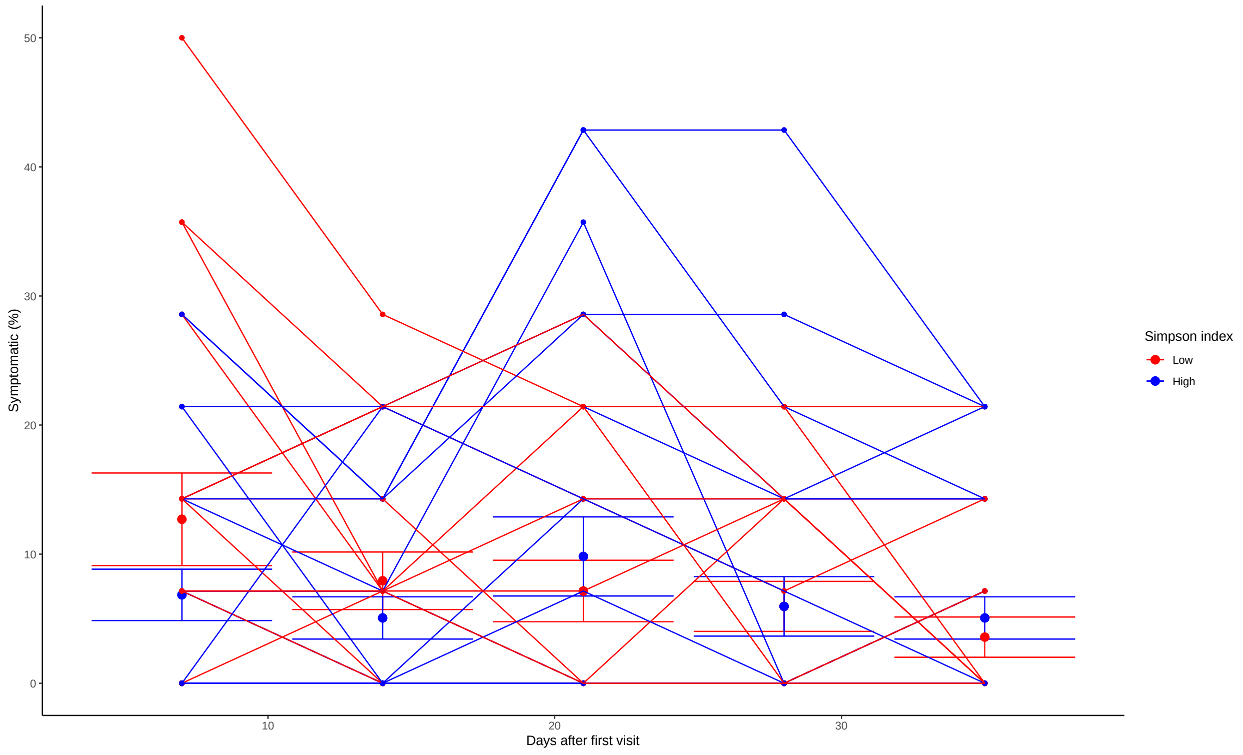

Supplemental Figure 5. Longitudinal analysis of symptom recovery and BMI within diversity groups. Pairwise regression models grouped by lower and upper diversity cut-offs. Percent BMI composition of lower diversity group: normal BMI (12.5%), overweight BMI (12.5%), obese BMI (75%). Lower diversity group coefficient estimates and p-values by BMI group: normal BMI (coefficient = -0.71), overweight BMI (coefficient = -6.43, $p<0.05$), and obese BMI (coefficient = -1.65, $p<0.01$). The model for normal BMI in the lower diversity group was not statistically significant. Percent BMI composition of upper diversity group: normal BMI (45%), overweight BMI (38%), obese BMI (17%). Upper diversity group coefficient estimates by BMI group: normal BMI (coefficient = 0.13), overweight BMI (coefficient = -1.11), and obese BMI (coefficient = 0.54). All models for the upper diversity group were not statistically significant.

BMI -> Age + Gender + Ethnicity + CRP + Leptin	Estimate	Std. error	Statistic	P-value
Intercept	26.14	5.440	4.806	<0.01
Age	-0.010	0.081	-0.102	0.919
Gender				
Female	0.750	2.647	0.283	0.779
Ethnicity				
NHPI	5.490	4.465	1.230	0.227
White	1.880	3.883	0.484	0.631
Other	1.760	5.145	0.342	0.735
Obesity biomarkers				
C-reactive protein (CRP)	0.041	0.017	2.426	0.021
Leptin	0.104	0.086	1.198	0.240
Leptin -> Age + Gender + Ethnicity	Estimate	Std. error	Statistic	P-value
Intercept	11.706	10.543	1.110	0.275
Age	0.035	0.157	0.222	0.826
Gender				
Female	-7.838	5.142	-1.524	0.137
Ethnicity				
NHPI	12.102	8.623	1.404	0.170
White	12.789	7.660	1.670	0.104
Other	5.406	9.925	0.545	0.590
CRP -> Age + Gender + Ethnicity	Estimate	Std. error	Statistic	P-value
Intercept	9.857	53.567	0.184	0.855
Age	0.581	0.798	0.728	0.471
Gender				
Female	-18.878	26.063	-0.724	0.474
Ethnicity				
NHPI	58.530	43.973	1.331	0.192
White	17.456	38.241	0.456	0.651
Other	80.861	50.666	1.596	0.120

Supplemental Table 1. Regression modeling of potential BMI covariates in our cohort. Default parameters set to male gender and Asian ethnicity. No independent variables significantly influence BMI.

Assignment	Sequencing number	SN2 number	Bases (MB)	>=Q20 bases	Reads	Mean read length (bp)	% >=Q20 bases
C006	506	5019	47309843	42490103	206219	229	89.8%
C009	512	7174	34723140	30970769	156186	222	89.2%
C010	513	7034	30475745	27219133	137688	221	89.3%
C008	514	7035	26819315	23958918	116412	230	89.3%
C013	517	7109	37369690	33078804	171404	218	88.5%
C014	518	7093	41409311	36981962	181793	227	89.3%
C015	519	7116	47241348	41872518	208476	226	88.6%
C016	521	7111	30639926	27390848	131070	233	89.4%
C022	524	7177	31220944	27881906	137047	227	89.3%
C020	525	6161	50065474	45237673	218459	229	90.4%
C021	526	6138	45504229	40467291	204081	222	88.9%
C019	528	6198	30534389	27331098	135823	224	89.5%
C018	529	6175	14126596	12593443	62175	227	89.1%
C026	530	6153	29992993	26771992	129037	232	89.3%
C030	531	7181	40634152	36243770	179187	226	89.2%
C029	532	6170	35164568	31464617	153721	228	89.5%
C027	533	6164	56685387	50990937	238119	238	90.0%
C031	536	7180	61414518	54845350	261149	235	89.3%
C032	537	6178	53613863	48120452	227499	235	89.8%
C033	538	6163	96879527	86932209	406973	238	89.7%
C034	539	6154	68568944	61704930	287416	238	90.0%
C036	541	7104	93120492	83938500	389562	239	90.1%
C037	542	7115	64386802	57999688	273920	235	90.1%
C038	543	7306	56995035	50481421	238536	238	88.6%
C040	545	7196	91065024	81288651	372856	244	89.3%
C041	546	7190	50570745	45287780	216343	233	89.6%
C043	547	7187	53482049	48449322	224401	238	90.6%
C044	548	7303	60503778	54704774	255246	237	90.4%
C047	551	7288	26398229	23815382	114981	229	90.2%
C048	552	7299	54551481	48690273	233897	233	89.3%
C049	553	7276	46218139	41763265	196852	234	90.4%
C051	555	7300	79482141	70862927	335060	237	89.2%
C053	557	7188	63279362	56875297	262662	240	89.9%
C054	558	7272	79944102	70871341	336349	237	88.7%
C055	559	7273	102152367	91906597	419166	243	90.0%
C056	560	7302	98024484	87414327	409250	239	89.2%
C059	563	7179	44784547	40399815	189910	235	90.2%
C060	564	7213	51677637	46630026	215969	239	90.2%
C062	565	7292	46471735	41836811	196644	236	90.0%
C063	566	7287	53930756	48464957	228685	235	89.9%
C065	568	7264	61717242	55326517	263772	233	89.6%
C066	569	7204	50794028	46071399	210751	241	90.7%
C067	570	7216	44784547	40399815	189910	235	90.2%
C070	573	7253	46471735	41836811	196644	236	90.0%
C068	575	7285	61717242	55326517	263772	233	89.6%
C061	576	7304	50794028	46071399	210751	241	90.7%
C073	578	7277	66267858	60039798	288873	229	90.6%
C072	579	7249	66200870	59787803	284789	232	90.3%
		**average**	**53670424**	**48147707**	**228531**	**233**	**89.7%**

Supplemental Table 2. Sequencing statistics of gut microbial DNA using Ion S5 Next-Generation Sequencing system with Ion 530 chips platform.

Data variables	R	P-value
Demographic		
Age	-0.23	0.133
Neutralization capacity		
Baseline	0.31	0.0345
Final follow-up	0.2	0.182
High Mobility Group Box 1		
Baseline	-0.23	0.112
Final follow-up	-0.31	0.0308
Gut microbiome (genus)		
Hyphomicrobiaceae	-0.45	0.003
Bifidobacteriaceae	-0.43	0.004
Cytophagaceae	0.40	0.009
Carnobacteriaceae	-0.39	0.011
Deferribacteraceae	0.38	0.012
Helicobacteraceae	0.37	0.015
Verrucomicrobiaceae	0.37	0.015
Thermoanaerobacteraceae	0.34	0.026
Ruminococcaceae	-0.34	0.030
Streptococcaceae	0.030	0.040
Peptostreptococcaceae	-0.31	0.044
Comamonadaceae	-0.31	0.0486

Supplemental Table 3. Correlation analysis used for deriving step-wise pathway.

	Estimate	Std. error	Z-value	P(>|Z|)
Indirect effect (mediation effect)	-3.554	5.365	-0.23	0.508
Total effect	-47.908	13.568	0.31	0

Supplemental Table 4. Mediation analysis outcome computed using Lavaan R package. No mediation effects were observed in the path analysis.
